# Hybrid Membranes of the Ureasil-Polyether Containing Glucose for Future Application in Bone Regeneration

**DOI:** 10.3390/pharmaceutics15051474

**Published:** 2023-05-12

**Authors:** Camila Garcia da Silva, João Rodrigues Monteiro, João Augusto Oshiro-Júnior, Leila Aparecida Chiavacci

**Affiliations:** 1 School of Pharmaceutical Sciences, São Paulo State University (UNESP), Araraquara 14800-903, SP, Brazil; 2 Graduate Program in Pharmaceutical Sciences, Biological and Health Sciences Center, State University of Paraiba (UEPB), Campina Grande 58429-500, PB, Brazil

**Keywords:** organic–inorganic hybrids, polypropylene oxide, polyethylene oxide, sol–gel process, glucose release, bone regeneration

## Abstract

The application of mesenchymal stem cells (MSC) in bone tissue regeneration can have unpredictable results due to the low survival of cells in the process since the lack of oxygen and nutrients promotes metabolic stress. Therefore, in this work, polymeric membranes formed by organic–inorganic hybrid materials called ureasil-polyether for modified glucose release were developed in order to overcome the problems posed by a of lack of this nutrient. Thus, membranes formed by polymeric blend of polypropylene oxide (PPO4000) and polyethylene oxide (PEO500) with 6% glucose incorporation were developed. Physical–chemical characterization techniques were performed, as well as tests that evaluated thermal properties, bioactivity, swelling, and release in SBF solution. The results of the swelling test showed an increase in membrane mass correlated with an increase in the concentration of ureasil-PEO500 in the polymeric blends. The membranes showed adequate resistance when subjected to the application of a high compression force (15 N). X-ray diffraction (XRD) evidenced peaks corresponding to orthorhombic crystalline organization, but the absence of glucose-related peaks showed characteristics of the amorphous regions of hybrid materials, likely due to solubilization. Thermogravimetry (TG) and differential scanning calorimetry (DSC) analyses showed that the thermal events attributed to glucose and hybrid materials were similar to that seen in the literature, however when glucose was incorporated into the PEO500, an increase in rigidity occurs. In PPO400, and in the blends of both materials, there was a slight decrease in Tg values. The smaller contact angle for the ureasil-PEO500 membrane revealed the more hydrophilic character of the material compared to other membranes. The membranes showed bioactivity and hemocompatibility in vitro. The in vitro release test revealed that it is possible to control the release rate of glucose and the kinetic analysis revealed a release mechanism characteristic of anomalous transport kinetics. Thus, we can conclude that ureasil-polyether membranes have great potential to be used as a glucose release system, and their future application has the potential to optimize the bone regeneration process.

## 1. Introduction

Bone repair is considered to be a regenerative process [1] that is complex and directly controlled by physiological and genetic factors [2]. However, bone regeneration is aggravated when the physiological process comes across critical bone defects [3,4].

Traditional grafting techniques for bone regeneration have limitations and disadvantages associated with the possible transmission of diseases or infections, immunological rejection by the recipient host, limited availability, two surgical fields (recipient and donor), donor site morbidity, pain, hemorrhage, loss of function, among others [5,6,7,8,9,10].

Thus, researches were developed as alternatives to assist bone tissue repair [11,12]. One of these alternatives is the use of mesenchymal stem cells (MSC), which have the ability to differentiate into different cell lines, thus also integrating tissue and bone regeneration [6]. Cells can be implanted in the injured site alone or associated with bioactive synthetic materials [13]. However, the use of MSC has unpredictable results [8,14], because the low cell survival rate (10 to 20%) makes the method inefficient [15,16].

This is explained by the ischemic environment (scarcity of oxygen and nutrients), caused by the absence of vascularization in the defects and the lack of oxygen, which was considered the most harmful factor affecting cell survival [16,17,18,19,20]. However, Deschepper et al. questioned this paradigm [18]. In vivo and in vitro results revealed that the addition of exogenous glucose (main nutrient for cellular metabolism) plays a critical role in the ischemic environment, allowing osteoprogenitor cells to survive in a near-anoxic environment (complete oxygen deprivation).

Thus, evidence shows that exogenous glucose acts to improve the survival and functionality of MSCs implanted in bone defects [18,20,21]. Therefore, the data suggest the importance of developing new strategies for the incorporation and release of exogenous glucose, with the objective of improving cell viability and bone tissue neoformation [20].

From the context of active release, therapy involving the use of controlled release systems is highlighted. In which, assets can be released over extended periods of time or to a specific target, so these systems have several advantages over traditional systems [22,23,24,25]. Materials aimed to improve tissue regeneration must have several requirements, including being biocompatible and mechanically resistant [26]. In addition, they must favor cell adhesion and migration, and promote the incorporation of bioactive molecules and/or nutrients, as well as control their release [12].

Thus, organic–inorganic hybrid polymeric materials have expanded in their development since the 1980s [27,28,29], and it is a unique material, formed by the mixture on a nanometric scale of organic (or biological) and inorganic components, synergistically combining the physicochemical characteristics of their components. This allows for the achievement of unique properties, making this class of materials excellent for the development of new multifunctional systems with broad applications, such as membrane separation processes, drug delivery systems, implants, and others [27,30,31,32,33].

Hybrid polymeric membranes called ureasil-polyether, which originated through the addition of organic polymers in silica networks formed through the sol–gel process [34], stand out because they have characteristics such as biocompatibility, high flexibility, thermal and mechanical resistance, and the ability to control the release of various substances. Therefore, they are able to act as a controlled release system for drugs and other biologically active substances [3,35,36,37]. In addition, the use of these materials in bone regeneration has advantages, since silica (SiO_2_) has biocompatible and osteoconductive properties, and the silanol groups (Si-OH) help in the formation of bone apatite, thus favoring the increase in the bioactivity of the materials [38].

Recently, the literature data have shown the possibility of improving some mechanical and drug delivery characteristics of hybrid membranes from the blends of hybrid precursors based on PEO and PPO with different molecular weights and proportions. For example, in vitro results revealed that the mixture, in the proportions of 80% ureasil-POE and 20% ureasil-POP, released 21% of the osteogenic growth peptide after 48 h and exhibited zero order release mechanisms. This is ideal for drug release since it allows for sustained active release in the same amount per unit time [31]. The flexibility of membranes can also be improved with the use of the polymeric blends to become more easily manageable during the implant process [3].

Therefore, this work aimed to develop ureasil-polyether hybrid membranes, containing polymeric blends of different molecular weights and chemical natures, to produce a material with the adequate characteristics to promote the controlled release of glucose for future application in bone regeneration, in order to improve the survival of MSC and increase the quantity and quality of bone tissue formed.

## 2. Materials and Methods

### 2.1. Preparation of the Ureasil-Polyether Hybrid Materials and Glucose Incorporation

The ureasil-polyether hybrid materials were synthesized using the sol–gel process, which has been well-described in the scientific literature [39,40]. First, the precursors were prepared from PEO500 (Sigma-Aldrich, São Paolo, Brasil > 99% purity, CAS #65605-36-9) and PPO4000 (Sigma-Aldrich, São Paolo, Brasil > 99% purity, CAS #9046-10-0) polymers, both dissolved in absolute ethanol [41]. Then, a modified alcooxide (ICPTES) (Sigma-Aldrich, São Paolo, Brasil > 95% purity, CAS #24801-88-5) was added, with a molar ratio of 1:2 (polymer/alcooxide), the quantities of reagents used are described in Table S1. The resulting solution was kept in a reflux condenser and stirred for 24 h at 75 °C and 400 rpm. Subsequently, the ethanol was eliminated by evaporation under vacuum at 80 °C and 175 mbar in a rotaevaporator (IKA RV 10, Staufen, Germany), forming the hybrid precursors.

To prepare the ureasil-polyether hybrid membranes, the hybrid precursor (0.750 g) was subjected to hydrolysis and condensation reactions. Hydrolysis and condensation were promoted by the addition of 1000 µL ethanol, 500 µL water, and 50 µL HCl catalyst (2M). Active glucose was incorporated in the proportion of 6%—0.045 g (mass/mass) in relation to the mass of the hybrid precursor, and it was dissolved during the hydrolysis and condensation step, together with ethanol and water. 

### 2.2. Assessment of Swelling

To understand the behavior of swelling caused by water absorption, pre-weighed dry membranes were immersed in simulated body fluid (SBF) solution under the conditions of 37 °C (±1.0 °C), and agitation of 50 rpm. At defined periods (0, 15, 30, 60, 120, 180, 240 and 360 min), the membranes were removed from the solutions, placed on absorbent paper to remove excess liquid, and then weighed on an analytical balance (Shimadzu, Barra Funda, Brazil). After weighing, the membranes were placed back into the solution. Swelling was evaluated from the percentage gain in mass of wet samples and calculated by the Equation (1):

(1) $$\%\;S=\frac{\left(m_{i}-m_{f}\right)}{\left(m_{f}\right)}\times 100$$

where *S* is the percentage swelling rate, *M_i_* e *M_f_* (*m_i_* e *m_f_*) are the masses (grams) of the dry samples initially and after immersion, respectively. The test was performed in triplicate.

The preparation of the standardized SBF solution was based on the standard ISO 23317:2007 [42].

### 2.3. Dynamic Mechanical Analysis (DMA)

The DMA technique was used to determine the mechanical strength of membranes when they were subjected to a variation in the compression force on their surface. The DMA 2980 equipment (TA-Instruments, New Castle, DE, USA) was used, operating at 37 °C, closed oven, with a power ramp of 1 N/min up to 15 N, compression load mode and cylindrical-shaped specimen with 6.25 mm diameter and 0.5 mm thickness.

### 2.4. X-ray Diffraction (XRD)

X-ray diffraction measurements were collected from the D5000 diffractometer (Siemens/Bruker, Aubrey, TX, USA), operated with Cu Ka radiation monochromated by graphite single crystal (k = 0.1542 nm), with a step of 0.05°/1 s, with an initial angle of 5° and an end of 80°. 

### 2.5. Differential Scanning Calorimetry (DSC)

The equipment used for thermal analysis measurements was the Q20 Calorimeter (DSC) (TA Instruments, New Castle, DE, USA). The curves were obtained at a temperature range of −75 °C to 350 °C, with a heating rate of 10 °C/min under nitrogen atmosphere (50 mL/min). The samples were cut in a circular shape and placed in hermetic 40 µL aluminum crucibles. 

### 2.6. Contact Angle Assessment (Wetability)

The contact angle of the membranes with water and the shape of the water droplets were measured at room temperature, under air atmosphere, by means of a Goniometer—OCA instrument coupled with a CCD camera with the aid of the SCA20 2.0 software (Dataphysics, Filderstadt, Germany). A drop of water (7 µL) was deposited on the membrane surface using a computer-controlled syringe and needle, and the air/water/membrane contact angle was determined by image analysis using the software described above. Each contact angle value was obtained from an average of three samples.

### 2.7. In Vitro Bioactivity of Ureasil-Polyether Membranes

Bioactivity was analyzed by immersing the hybrid membranes in plastic bottles with a lid containing SBF solution and kept in a water bath at 37 °C for 12 h, 24 h, 3 days, 7 days and 14 days, where the surface area/volume ratio was 0.1 cm^−1^ (ISO 23317/2007) [42]. The solution was not replenished for the entire test period [43]. After the defined periods, the membranes were removed from the solution, washed carefully with deionized water and dried at room temperature in a vacuum desiccator for approximately 12 h.

Subsequently, the material was covered by evaporation of 1 carbon wire, generating a coverage of approximately 5 nm thick, and the membrane surfaces were evaluated by scanning electron microscopy technique (SEM) in Scanning Electron Microscope JSM7500F (JEOL, Musashino, Akishima, Tokyo, Japan), and then characterized by energy dispersion X-ray spectroscopy (EDS), coupled with SEM.

To assess the pH changes induced by the membranes, the pH of the SBF solution collected at the end of each evaluated time was checked, using a digital pH meter B 500 (ION, Araucaria, Brazil).

### 2.8. Assessment of Hemolysis Potential

The experimental procedure was conducted with the approval of the Ethics Committee of the State University of Paraíba—UEPB (CAAE 52812121.1.0000.5187). Healthy adult volunteer donors, aged between 18 and 50 years, of both sexes and carriers of different blood types, were recruited. Blood samples were collected in tubes containing EDTA. The quantification of hemoglobin released by hemolysis is based on the method described by BAJPAI et al. (2015) and CHHATRI et al. (2011), which made adaptations to the methodology based on recommendations by the American Society for Testing and Materials—ASTM (ASTM F756) [44,45].

Briefly, plasma was separated from blood samples by centrifugation for 5 min at 2000 rpm and discarded. The suspension was washed twice with 0.9% NaCl (1:1) and centrifuged to obtain erythrocytes. The sediment from the second centrifugation was suspended in 0.9% NaCl to obtain 0.5% suspension of blood. Then, to assess the possible hemolysis potential, the sample (1 cm^2^) was incubated in 7 mL 0.9% NaCl at 37 °C for 24 h, the saline solution was removed, and 50 µL of the erythrocyte concentrate was added to the surface of the sample. It was slowly shaken for greater contact of the suspension with the sample and remained in contact for 15 min. A total of 10 mL NaCl 0.9% was added and incubated for 3 h at 37 °C. After incubation, the fluid was transferred to a test tube and centrifuged at 104 rpm for 15 min. Finally, the hemoglobin released by hemolysis was measured by the absorbance of the supernatant at 545 nm using a UV-1900 spectrophotometer (Shimadzu, Kyoto, Japan). A total of 50 µL of the erythrocyte suspension + 10 mL of deionized water was used as a positive control, and 50 µL of the erythrocyte suspension + 10 mL of 0.9% NaCl as a negative control. The experiments were performed in triplicate. The percentage of hemolysis was calculated by the following Equation (2):

(2) $$\%Hemolysis=\frac{\left({Abs}_{sample}-{Abs}_{control\;neg.}\right)}{\left({Abs}_{control\;pos.}-{Abs}_{control\;neg.}\right)}\times 100$$

where:

Abs sample (*Abs_sample_*) = sample absorbance;

Abs control neg. (*Abs_control neg_*) = absorbance of the negative control;

Abs control pos. (*Abs_control pos_*) = absorbance of the positive control.

### 2.9. In Vitro Glucose Release from Ureasil-Polyether Membranes

Membranes containing 6% (0.045 g) of glucose were immersed in 100 mL of simulated body fluid (SBF) solution under conditions of 37 °C (±1.0 °C), with an agitation of 50 rpm, and pH 7.4. To collect each of the aliquots, a micropipette was used. The volume collected from the aliquots was 2 mL. This was replaced soon after to keep the sink conditions. Aliquots were collected and analyzed at selected time intervals in 15 min, 30 min, 1 h, 2 h, 3 h, 4 h, 6 h, 12 h, 24 h (1 day), 48 h (2 days), 72 h (3 days), 96 h (4 days), 168 h (7 days), 264 h (11 days), and 336 h (14 days).

Glucose quantification was performed by a specific enzymatic system for glucose determination (Labtest, Liquiform Glucose—Lagoa Santa, Brazil), where the methodology used is described in the instructions for use of the enzyme kit [46]. In a test tube, 10 μL of the sample and 1 mL of the reagent were added, kept incubated in a water bath at 37 °C for 10 min, and subsequently, the absorbance reading was performed in a Power Wave XS2 plate spectrophotometer (Biotek, Winooski, VT, USA). The cumulative asset release percentages were calculated from the average of three different monitorings. The results were expressed as the mean ± SE of the triplicate.

The asset release kinetics were evaluated by applying different mathematical models of kinetics [47,48], with the aid of Sigma Plot 10 software (Systat Software Inc., San Jose, CA, USA).

## 3. Results and Discussion

### 3.1. Preparation of the Ureasil-Polyether Hybrid Materials and Glucose Incorporation

The ureasil-polyether hybrid membranes were obtained after drying the gel formed after the hydrolysis and condensation reactions of the hybrid precursors under vacuum in a desiccator at room temperature, in approximately 13 days. Membranes were developed from polymeric blends called ureasil-PEO500/PPO4000 in different proportions (mass/mass).

**Figure 1:**
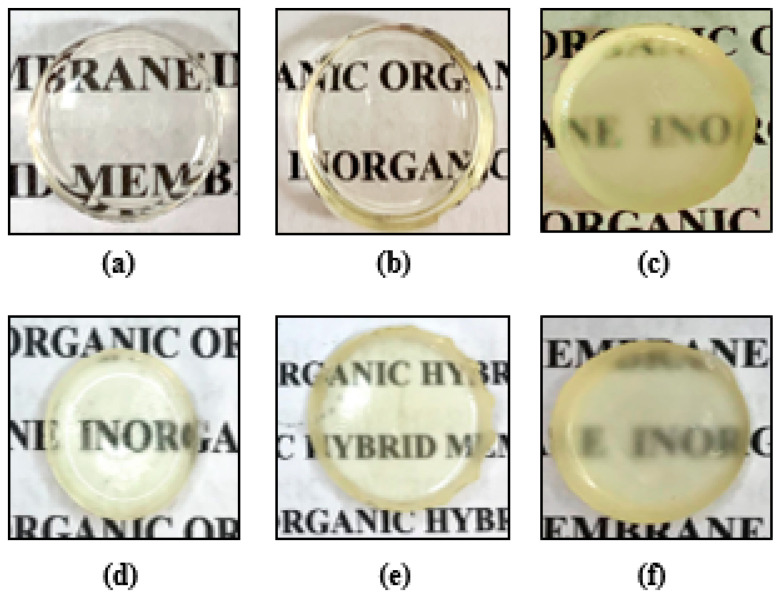
Photographs of the visual appearance of membranes without glucose and containing 6% glucose. Unloaded glucose membranes ureasil-PEO500 (**a**), ureasil-PPO4000 (**b**), and ureasil-PEO500/PPO4000 in the proportion 50/50 (**c**). Loaded 6% glucose membranes ureasil-PEO500 (**d**), ureasil-PPO4000 (**e**), and ureasil-PEO500/PPO4000 in the proportion 50/50 (**f**).

The ideal concentration of glucose incorporated in the formation of hybrid membranes was determined according to tests performed at different concentrations, and the maximum concentration of glucose that could be incorporated without visually altering the homogeneity of the membranes was 6% (0.045 g) of glucose, since macroscopically there is a uniform dispersion/dissolution within the hybrid matrix, without causing irregularities (broken and/or cracks) or precipitates on the surface, both for membranes prepared from individual precursors, or blends of them (Figure 1). These characteristics were observed during 3 months of storage. Therefore, this method helped in the solubilization and caused the glucose to be retained and/or dispersed in the polymer matrix, possibly, at one of the interaction sites of the ureasil-polyether membrane, such as silanol, urea and ether-type oxygen atoms [28,49]. 

### 3.2. Assessment of Swelling

**Figure 2:**
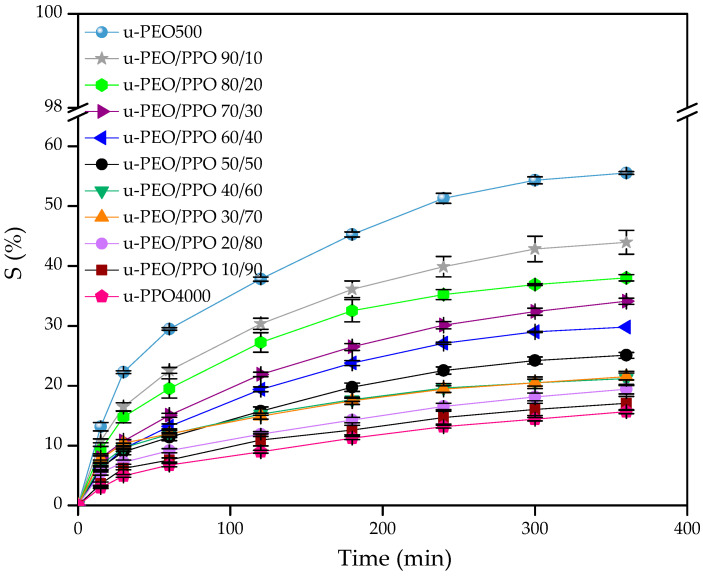
Percentage of swelling (S) as function of immersion time in SBF of loaded 6% glucose membranes prepared from individual precursors and from blends of ureasil-PEO500/PPO4000.

The swelling profile of the membranes was determined to help choose the best proportions of the polymeric blends used. The evolution of the increase in mass, associated with swelling, of the hybrid membranes can be seen in Figure 2.

The increase in mass, and consequently the swelling of the membranes, occurs with the increase in the concentration of ureasil-PEO500 in the blend and in the membrane ureasil-PEO500. The results showed that for the ureasil-PEO500 membrane the swelling was ≈55%, and for membrane ureasil-PPO4000 ≈ 16%. For the blends, the results were ≈44% for ureasil-PEO500/PPO4000 90/10, ≈17% for the 10/90 proportion, and ≈25% for the 50/50 proportion. The difference in swelling between the membranes occurs because the ureasil-PPO4000 hybrid has a more hydrophobic character due to the support exerted by CH3 groups linked to ether-type oxygen, a fact that does not occur in the ureasil-PEO500 hybrid [50]. The more hydrophobic character of the ureasil-PPO4000 hybrid makes the membranes show less affinity for the SBF solution and, therefore, decreases its swelling capacity in relation to ureasil-PEO500 membrane.

It is noted that the different proportions in the polymeric blends can change the swelling profile, attributing an intermediate degree of swelling when compared to membranes prepared from individual precursors. Consequently, the different proportions can also modulate the rate of glucose release, in addition to preventing the occurrence of trauma in vivo, which could lead to a marked inflammatory response. As reported in the literature, for membranes prepared from the precursors ureasil-PEO1900 and ureasil-PPO400, in which the ureasil-PEO1900 membrane showed greater swelling, causing acute inflammation. On the other hand, the ureasil-PPO400 membrane did not show tissue damage after 15 days of implantation, possibly due to less swelling [3]. Thus, to select the proportions of the membranes tested, data from the literature were used as a reference, which showed a result of swelling of the ureasil-PPO400/PEO1900 (80/20) as 14.87% after 60 min of experiment, and satisfactorily presented biocompatibility in the in vivo test [3]. Therefore, the selected blends of this work are the proportions with swelling values close to the reference (14.87%), referring to the membranes ureasil-PEO500/PPO4000—20/80 (≈9%), 30/70 (≈12%), 40/60 (≈12%), 50/50 (≈12%) and 60/40 (≈14%).

### 3.3. Dynamic Mechanical Analysis (DMA)

**Figure 3:**
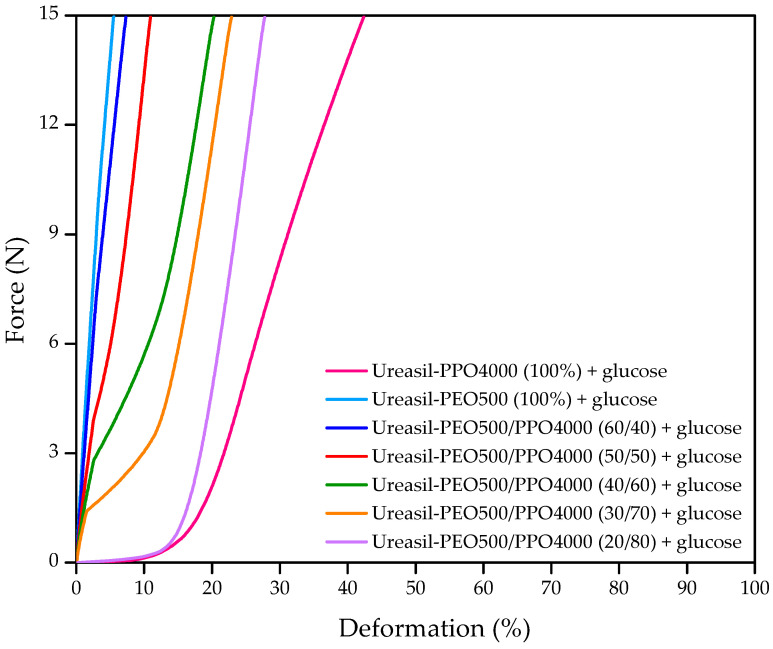
Strain curves as a function of applied force to loaded 6% glucose membranes prepared from individual precursors and from blends of ureasil-PEO500/PPO4000.

Figure 3 shows the deformation curves due to the compression force (15 N) applied to the membranes.

The deformations were approximately 6% for ureasil-PEO500 membranes, and 42% for ureasil-PPO4000 membranes. Ureasil-PEO500 membranes are more rigid due to a polymer chain with lower molecular weight that has higher numbers of crosslinking nodes, therefore, they are more resistant to deformation [51]. On the other hand, ureasil-PPO4000 membranes with higher molecular weight are more flexible and have lower resistance to deformation. Therefore, among the blends of precursors, in membranes prepared with larger amounts of the precursor ureasil-PPO4000, there is a steady incline in percentage of deformation. For larger amounts of ureasil-PEO500, the deformation is smaller. Among the blends of precursors, the more flexible membrane (presenting the highest value of ≈28% deformation) was ureasil-PEO500/PPO4000—20/80.

In the evaluation of the mechanical resistance to compression, membranes prepared from the blends ureasil-PEO500/PPO4000 presented values of mechanical resistance to compression between 0.25 MPa and 0.33 MPa. These results are in agreement with the results of mechanical resistance to compression in materials intended for bone regeneration, which present values between 0.013 MPa and 0.55 Mpa (MPa) [52,53,54,55].

The DMA results showed that the mechanical properties of the analyzed membranes could be modulated, just as it occurs in swelling behavior. Furthermore, despite the large applied compression force, the membranes did not show rupture or any macroscopic damage, emphasizing their good mechanical resistance.

### 3.4. X-Ray Diffraction (XRD)

**Figure 4:**
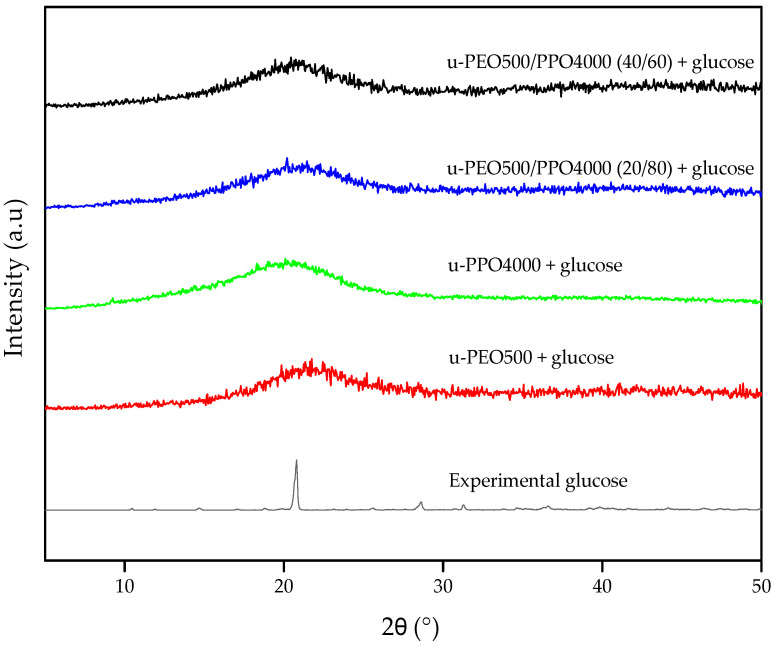
X-ray diffraction patterns of experimental glucose and loaded 6% glucose membranes prepared from individual precursors and from blends of ureasil-PEO500/PPO4000.

Figure 4 shows the X-ray diffractograms of glucose incorporated in hybrid membranes (called experimental glucose), membranes prepared from individual precursors and membranes prepared from the blend of precursors in the defined proportions according to the evaluation of swelling and mechanical resistance, all containing glucose.

The glucose diffraction pattern is identified by fine, high-intensity peaks, suggesting a crystalline phase. The peaks present in the experimental glucose diffractogram are in accordance with the pattern found for the compound D-glucose, which has an orthorhombic crystalline system and space group P2_1_2_1_2_1_, in CIF 1518432 [56].

For all analyzed membranes, we can observe only the presence of a broad peak, representative of the inorganic amorphous siloxane regions of the ureasil-polyether hybrids [57,58]. It is evident that the fine and well-defined peaks associated with glucose are not present in the hybrid membranes, regardless of whether they are formed by individual or blend ureasil-PEO and ureasil-PPO. This fact may have happened because glucose was solubilized when incorporated into the polymer matrix. This hypothesis corroborates the visual appearance of the membranes obtained after the incorporation of glucose.

### 3.5. Differential Scanning Calorimetry (DSC)

**Figure 5:**
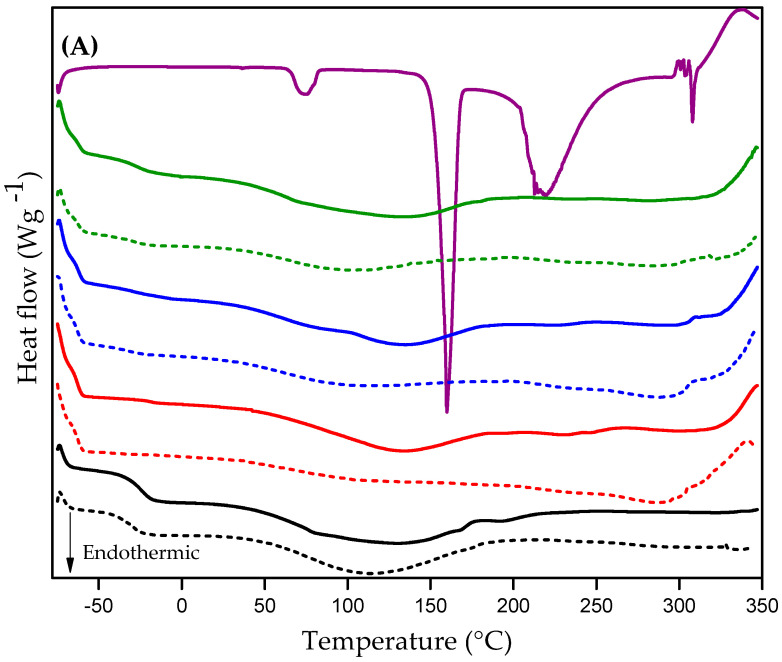

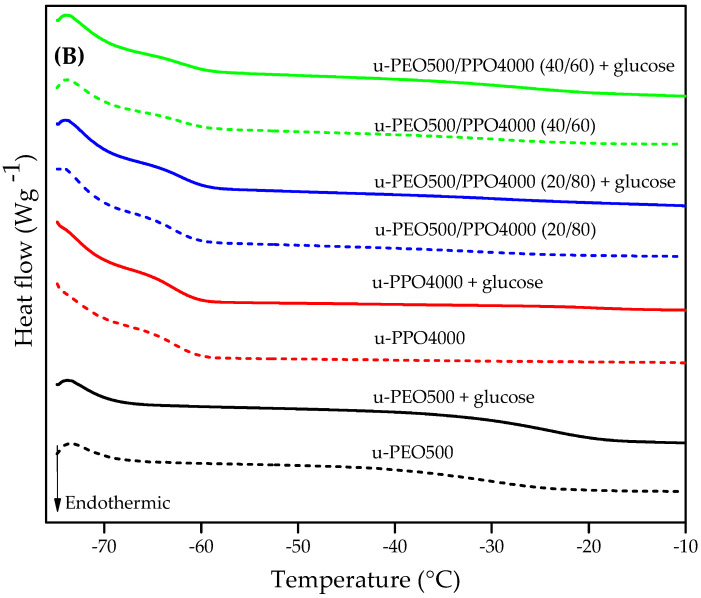
(**A**) DSC curves of glucose (purple curve), ureasil-PEO500 (dashed black curve), ureasil-PEO500 loaded glucose (black curve), ureasil-PPO400 (dashed red curve), ureasil-PPO laoded glucose (red curve), blend of ureasil-PEO500/PPO400 (20/80) (dashed blue curve), blend of ureasil-PEO500/PPO400 (20/80) loaded glucose (blue curve) and blend of ureasil-PEO500/PPO400 (40/60) (dashed green curve), blend of ureasil-PEO500/PPO400 (40/60) loaded glucose (green curve). (**B**) is a magnification of the DSC curves of the glass transition temperature 351 (Tg) region.

Changes in thermal events caused by asset incorporation can provide information about asset-matrix interactions. Figure 5A displays the DSC curves recorded on glucose (black curve), ureasil-PEO500 (dashed blue curve), ureasil-PEO500 loaded glucose (blue curve), ureasil-PPO400 (dashed pink curve), ureasiL-PPO laoded glucose (pink curve), blend of ureasil-PEO500/PPO400 (20/80) (dashed purple curve), blend of ureasil-PEO500/PPO400 (20/80) loaded glucose (purple curve) and blend of ureasil-PEO500/PPO400 (40/60) (dashed green curve), blend of ureasil-PEO500/PPO400 (40/60) loaded glucose (green curve). Figure 5B highlights the temperature range between −70 and −10 °C for all materials to show the glass transition temperature (Tg).

We can see in Figure 5 that the experimental glucose DSC curve shows an endothermic peak at 75 °C equivalent to glucose dehydration, a melting peak at 160 °C, accompanied by two endothermic peaks at 219 °C and 308 °C, and an exothermic peak at 332 °C related to the active decomposition steps. The melting temperatures of sugars do not show a consensus in the literature as, for glucose, the temperature values vary between 146 and 165 °C [59,60,61], but there are reports of decomposition temperature above 200 °C [60].

In all of the membranes we analyzed, the glass transition temperatures showed negative values, as described in Table S2. Negative Tg values are associated with the high flexibility characteristic of polymers. Therefore, the closer to zero the Tg value, the greater the rigidity of the polymer chain [62].

Ureasil-PEO500 membranes, with or without glucose, presented results closer to zero, thus indicating greater rigidity compared to the others. This fact may be associated with the higher number of crosslinking nodes due to the polymer chain of ureasil-PEO500 membranes having a lower molecular weight. Among the membranes prepared from the blends of precursors, we observed a slight increase in the Tg value (closer to zero) when the proportion of ureasil-PEO500 is increased.

We observed that the Tg value increased from −30.40 °C to −23.90 °C when glucose was incorporated into the ureasil-PEO500 membrane, indicating an increase in rigidity of the membrane. On the other hand, there was a slight decrease in Tg values when glucose was incorporated into the membrane ureasil-PPO4000, and in the blends. This behavior suggests that glucose molecules may not significantly affect the rigidity of PPO polymer chains. Similar results were reported by LOPES et al. (2012) when the drug diclofenac sodium was added in the ureasil-PEO1900 and ureasil-PEO500 matrices, and by MOLINA et al. (2013) in the incorporation of ibuprofen in Jeffamine^®^ T-5000 (PPO) [49,63].

In the region between 80 °C and 180 °C, there is a broad endothermic peak in all membranes, related to the presence of residual solvents remaining from the synthesis of the material. The exit of water is normally observed at temperatures below 100 °C; however, entrapment within the polymeric network may occur, causing an increase in the exit temperature [64].

It is observed that the characteristic of glucose, as its endothermic peak is located at 75 °C and melting peak at 160 °C, are not present in the hybrid glucose loaded membranes, regardless of whether they are formed individually or by a blend of ureasil-PEO and ureasil-PPO. This could be due to the fact that glucose was completely solubilized when incorporated into the polymeric matrix, as seen in the XRD results.

The lack of melting peak in the DSC curves of all tested membranes reveals the amorphous characteristic of the materials, corroborating the XRD results. Furthermore, we also highlight the high thermal stability of the membranes, due to the absence of peaks related to material decomposition.

### 3.6. Contact Angle Assessment (Wetability)

**Figure 6:**
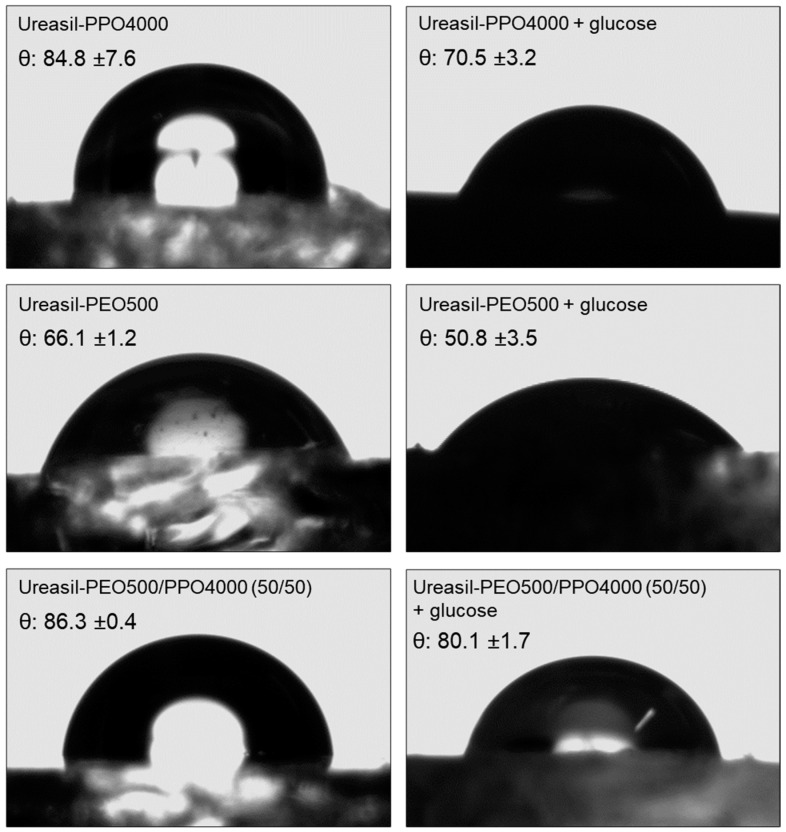
Contact-angle measurements of loaded 6% glucose membranes and unloaded glucose membranes prepared from individual precursors and from a blend of ureasil-PEO500/PPO4000 50/50. The results are expressed as the mean ± S.E. for n = 3 (replicates).

The images and information presented in Figure 6 allow us to compare the difference in the scattering of the water drop on the surface of the ureasil-polyether membranes and their respective contact angle values.

It is observed that the smallest spreading over the surface occurred in the membrane prepared from the blends of precursors ureasil-PEO500/PPO4000—50/50 without glucose, which resulted in a semi-spherical droplet with a mean contact angle value close to 90°, as well as for the membrane ureasil-PPO4000 without glucose. On the other hand, the ureasil-PEO500 membrane without glucose had an average contact angle of ≈66°.

For the ureasil-PEO500 membrane the water contact angle is much smaller and the droplet spread is much larger than that measured for the membranes obtained from the precursor PPO4000. This happens because the polymer PPO4000 has a more hydrophobic character than the polymer PEO500, where this high hydrophobicity repels the drop of water, which contracts its surface in an attempt to reduce as much as possible the contact with the membrane [50,65]. Furthermore, the literature says that surfaces with contact angles greater than or close to 90° are considered highly hydrophobic [66,67].

The results of contact angles for membranes containing glucose were on average ≈71° for ureasil-PPO4000, ≈51° for ureasil-PEO500, and for the membrane prepared from the blends of precursors ureasil-PEO500/PPO4000—50/50 was of ≈ 80°; therefore, all membranes showed a decrease in the contact angle, and consequently, an increase in hydrophilicity when compared to membranes without the presence of glucose. Thus, we can suggest that this result is associated with the hydrophilic nature of glucose, that is, it is an indication of the increase in hydrophilic groups on the surface of the membranes [68,69].

### 3.7. In Vitro Bioactivity of Ureasil-Polyether Membranes

There are reports in the literature that a large part of bioactive materials can form an apatite layer on their surface when in contact with the SBF solution in vitro for a time. Depending on the material, this time can vary from 1 to 3 days, or more [70]. Thus, through this layer, a strong chemical bond between the bioactive material (ions) and the bone increases in vivo [71], with the possibility of favoring the healing process in the entire bone defect. 

**Figure 7:**
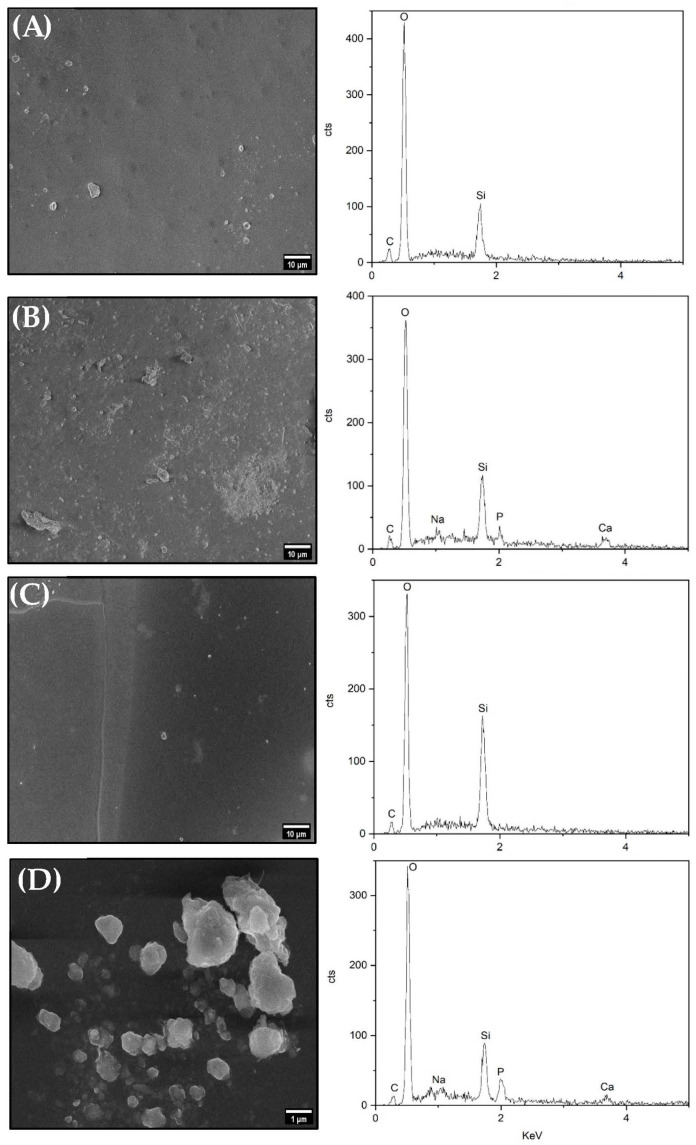
Scanning electron microscopy images and EDS spectra of loaded 6% glucose membranes surfaces after immersion in SBF solution. (**A**) ureasil-PEO500/PPO4000 (20/80) after 12 h. (**B**) ureasil-PEO500/PPO4000 (20/80), 14 days. (**C**) ureasil-PEO500/PPO4000 (40/60), 12 h. (**D**) ureasil-PEO500/PPO4000 (40/60), 14 days.

The microscopy images of the evaluation of the deposition of apatite on the surface of the membranes after immersion in SBF at the defined times, its morphology and the qualitative elemental analysis through EDS spectra are shown in Figure 7.

All membranes have a smooth surface, however, surface changes with precipitate depositions can be noted. From the evolution of the immersion time in SBF, a gradual increase in the number of precipitates and aggregates was observed. We can highlight the images and spectra of the membrane surfaces after immersion for 7 and 14 days in the two membranes analyzed, where the number of precipitates and aggregates are greater and indicate the presence of peaks the elements calcium (Ca) and phosphorus (P) in the EDS spectra. This peaks are characteristic of the components of hydroxyapatite (Ca_10_(PO_4_)_6_(OH)_2_), thus suggesting the formation of the hydroxyapatite layer [72,73,74], and similar observations were reported by BIGHAM et al. in 2020, SABER-SAMANDARI et al. in 2016, and SUTHA et al. in 2013 [75,76,77]. The elements carbon (C), oxygen (O) and silicon (Si) characteristic of ureasil-polyether hybrid membrane are also observed [51]. 

Due to the material’s ability to lead to the formation of an apatite nuclei layer, the continuous process of calcium and phosphate ion consumption present in the SBF solution contributes to the spontaneous growth of apatite on the surface [76,78,79].

Research demonstrates the importance of the presence of functional groups in bioactive materials, for apatite nucleation to occur [70]. There are reports that the existence of Si-OH, SiO_2_, and Na_2_O groups in the materials contribute to the formation of hydroxyapatite on their surface [72,80,81]. The Si element is recognized for its ability to act in the formation of apatite [82], and the advantages of using Si are directly related to its involvement in bone mineralization [78,81].

With the results obtained in this in vitro analysis, we can say that there are indications that the tested ureasil-polyether membranes are bioactive, that is, they are capable of favoring the connection between the material and the bone tissue from the formation of the hydroxyapatite layer on the surface of the material. This can be used in the future as a good inducer of bone regeneration.

The verification of the pH variation is extremely important for the biocompatibility of the material, since sudden changes can cause damage to the cells [83]. The ureasil-PEO500/PPO4000—20/80 and 40/60 membranes containing glucose showed similar results during the 14 days tested as the pH values of the SBF solution were between 7.44 and 7.56. The pH range known to be suitable for the cells is between 7.0 and 7.8 [84]. Thus, it is concluded that the membranes did not change the pH value of the SBF solution to values different from those acceptable.

### 3.8. Assessment of Hemolysis Potential

According to the norm ISO 10993-4 [85], any biomaterial or medical device whose biomedical application presents intimate and continuous contact with blood needs an assessment of its in vitro hemocompatibility. The hemolysis test allows for the evaluation of the integrity of erythrocytes after contact with the biomaterial or medical device under analysis. In compliance with the standard ASTM F756—00:2000—Standard Practice for Assessment of Hemolytic Properties of Materials from the American Society for Testing and Materials, a result of less than 2% in the hemolysis test means that the material is not considered hemolytic. If it is between 2 and 5%, the material is considered relatively hemolytic, but if it is greater than 5%, the material is hemolytic. 

The data obtained in the evaluation of the hemocompatibility of the ureasyl-polyether membranes containing 6% glucose (Figure S1) revealed that the membranes with the blends of precursors (50/50) and ureasil-PEO500 membrane showed no hemolytic behavior (≤2%) for blood groups A and B. Blood group O were considered relatively hemolytic (2–5%), as was the case for the ureasil-PPO4000 membrane (2–5%), which was considered relatively hemolytic for all blood types tested. The differences found in the rates of hemolysis between the blood groups can be explained by the absence or presence of specific antigens between the red blood cells of the different blood groups of the ABO classification system, which may cause different interactions and processes [86]. The results demonstrated that the hybrid membranes of the ureasil-polyether is hemocompatible (less than 5% hemolysis), according to the ASTM F756 [87,88,89]. These results corroborate previous in vitro cell viability studies using the MTT method and indicate biocompatibility when implanted in rat subcutaneous tissue.

### 3.9. In Vitro Glucose Release from Ureasil-Polyether Membranes

**Figure 8:**
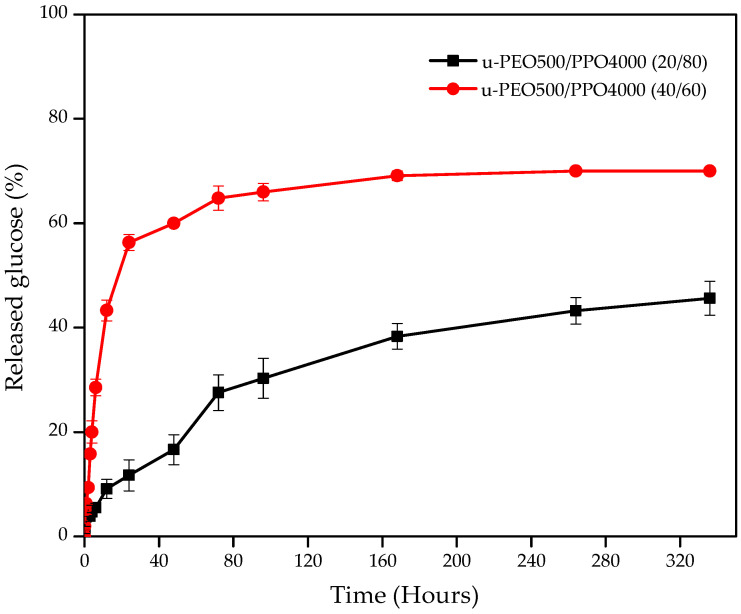
Glucose release (%) in loaded 6% glucose membranes prepared from blends of ureasil-PEO500/PPO4000—20/80 and 40/60 as a function of immersion time in SBF, for 15 min, 30 min, 1 h, 2 h, 3 h, 4 h, 6 h, 12 h, 24 h (1 day), 48 h (2 days), 72 h (3 days), 96 h (4 days), 168 h (7 days), 264 h (11 days) and 336 h (14 days).

Figure 8 shows the release curves (percentage of released glucose) of membranes prepared from the blends of glucose-containing precursors, at defined times.

It is observed that in both samples there is a gradual increase in the glucose released into the medium within approximately 168 h (7 days) of the experiment. After this period, the curves verifiably reach a plateau, especially for the membrane ureasil-PEO500/PPO4000—40/60, indicating that possibly the steady state equilibrium of the release was or is close to being reached [90].

Note that for the membrane prepared from the blends of precursors ureasil-PEO500/PPO4000—40/60 in the first 15 min of the experiment, ≈1.9% of glucose was released, while for the membrane ureasil-PEO500/PPO4000—20/80 the release had not yet started (0%). At the end of the 14 days of evaluation, the membrane with the highest proportion of ureasil-PEO500 released a greater amount of glucose (70.5%), when compared to the proportion with a smaller amount of ureasil-PEO500, which released 45.6%.

Higher concentrations of ureasil-PEO500 showed the highest swelling profile. Therefore, in this case, greater release of the incorporated active is expected, through the rapid penetration of SBF into the matrix. This is due to the hydrophilicity characteristic of the polymeric chair of ureasil-PEO500, as discussed above. Therefore, there is a relationship between swelling and the release profile, and it is also possible to modulate the active release profile through the nature of the polymer, such as the hydrophilic/hydrophobic balance of the blends of precursors [35,51,91].

For a better understanding of the asset transport mechanism, it was necessary to know the process release kinetics. The mathematical model that best suited the glucose release curves from the hybrid membranes tested was the one proposed by Korsmeyer–Peppas [92,93], through the Equation (3):

(3) $$\frac{M_{t}}{M_{\infty }}=k\cdot t^{n}$$

where: *M_t_* = Cumulative amount of drug released over time (*t*); *M*_∞_ = Total amount of drug released in an infinite time; *k* = Kinetic constant of release that considers structural and geometric characteristics of the system; *t* = Time; *n* = Release exponent that determines the release mechanism according to the numerical value.

However, the parameters of this equation can only be applied until 60% of total asset release occurs, where the percentage equation is valid [92,94]. In the Korsmeyer–Peppas model, the asset release transport mechanism is defined from the value of the exponent “*n*”, also considering the geometry of the system. In this case, cylindrical systems that swell were evaluated.

Table S3 shows the values of the coefficient of determination (r^2^), the values of the exponent “*n*”, using the Korsmeyer–Peppas equation, and the classification of the mechanism of glucose release through the tested membranes.

The values of the exponent “*n*” of the two proportions tested were between 0.45 and 0.89, 0.48 being for ureasil-PEO500/PPO4000—20/80 and 0.47 for ureasil-PEO500/PPO4000 40/60. Therefore, the release process occurs through a combination of the active diffusion mechanisms (Fickian diffusion) and controlled transport through swelling through polymer chain relaxation (Transport case II) [93].

## 4. Conclusions

Hybrid membranes were developed from a blend of different proportions of precursors ureasil-PEO500 and PPO4000 with the incorporation of 6% glucose without causing visual changes in the membranes. The difference in the hydrophilic–hydrophobic balance between the blends proportions made it possible to modulate the swelling characteristics, mechanical strength, flexibility, and glucose release.

The membranes showed mechanical resistance, thermal stability, absence of crystallinity and maintenance of the characteristic amorphous character of ureasil-polyether hybrids, in vitro bioactivity, hemocompatibility, and mainly, were able to incorporate and release glucose in a controlled manner.

Thus, as a key point, we can conclude that, for the first time in the literature, a hybrid membrane has been successfully developed using the ureasyl-polyether materials that utilizes glucose as a key component to aid the survival of mesequence cells and promote bone regeneration. Previously published data associated with hemocompatibility studies demonstrate that this membrane is biocompatible. Furthermore, the sol–gel production method allows for industrial scale-up, since the production cost is cheap.

Thus, this study represents an important advance in the field of materials engineering and healthcare that seeks innovative solutions for the regeneration of large bone defects. We hope that the hybrid membrane can be used in future clinical studies to promote bone regeneration in patients or inspire future studies that focus on materials that can lead to improvements in the quality of life of patients with bone injuries.

## Data Availability

Not applicable.

## Supplementary Materials

The following supporting information can be downloaded at: https://www.mdpi.com/article/10.3390/pharmaceutics15051474/s1, Table S1: Quantities of reagents used in the synthesis of precursors; Table S2: Tg values (°C) obtained from the DSC curves for the hybrid membranes; Table S3: Information obtained through the Korsmeyer-Peppas equation for the glucose release curves from the hybrid membranes; Figure S1: Comparison of the percentage of hemolysis between loaded 6% glucose mem-branes, ureasil-PEO500 and PPO4000, and blend of ureasil-PEO500/PPO4000 (50/50).
