# Supplementary material for: Hybrid Membranes of the Ureasil-Polyether Containing Glucose for Future Application in Bone Regeneration
Source: Pharmaceutics. 2023 May 12;15(5):1474. doi: 10.3390/pharmaceutics15051474 (PMC10223991; doi:10.3390/pharmaceutics15051474)
Supplement: Supplementary file 1 [file pharmaceutics-15-01474-s001.zip › pharmaceutics-2159358-supplementary.pdf]

pharmaceutics

Supplementary Materials

Hybrid Membranes of the Ureasil-Polyether Containing Glucose for Future Application in Bone Regeneration

Camila Garcia da Silva [1], João Rodrigues Monteiro [1], João Augusto Oshiro-Júnior [2] and Leila Aparecida Chiavacci [1,*]

[1] School of Pharmaceutical Sciences, São Paulo State University (UNESP), Araraquara 14800-903, SP, Brazil
[2] Graduate Program in Pharmaceutical Sciences, Biological and Health Sciences Center, State University of Paraiba (UEPB), Campina Grande 58429-500, PB, Brazil
* Correspondence: leila.chiavacci@unesp.br

Table S1. Quantities of reagents used in the synthesis of precursors.

Precursor	*ICPTES*	*Polymer*	*Ethanol*
PEO500	21.67 mL	25 g<br>(PEO - molecular weight 500 g.mol^{-1})	50 mL
PPO4000	3.26 mL	25g<br>(PPO - molecular weight 4000 g.mol^{-1})	50 mL

Table S2. Tg values (°C) obtained from the DSC curves for the hybrid membranes.

Material	*Tg (°C)*
Ureasil-PEO500 (100%)	- 30.40
Ureasil-PEO500 (100%) + 6% glucose	- 23.90
Ureasil-PPO4000 (100%)	- 62.80
Ureasil-PPO4000 (100%) + 6% glucose	- 62.92
Ureasil-PEO500/PPO4000 (20/80)	- 62.82
Ureasil-PEO500/PPO4000 (20/80) + 6% glucose	- 62.99
Ureasil-PEO500/PPO4000 (40/60)	- 62.20
Ureasil-PEO500/PPO4000 (40/60) + 6% glucose	- 62.34

Table S3. Information obtained through the Korsmeyer-Peppas equation for the glucose release curves from the hybrid membranes.

Material	r^2	*n*	*Release mechanism*
Ureasil-PEO500/PPO4000 (20/80) + 6% glucose	0.9806	0.48	Anomalous transport (non-Fickian)
Ureasil-PEO500/PPO4000 (40/60) + 6% glucose	0.9357	0.47	Anomalous transport (non-Fickian)

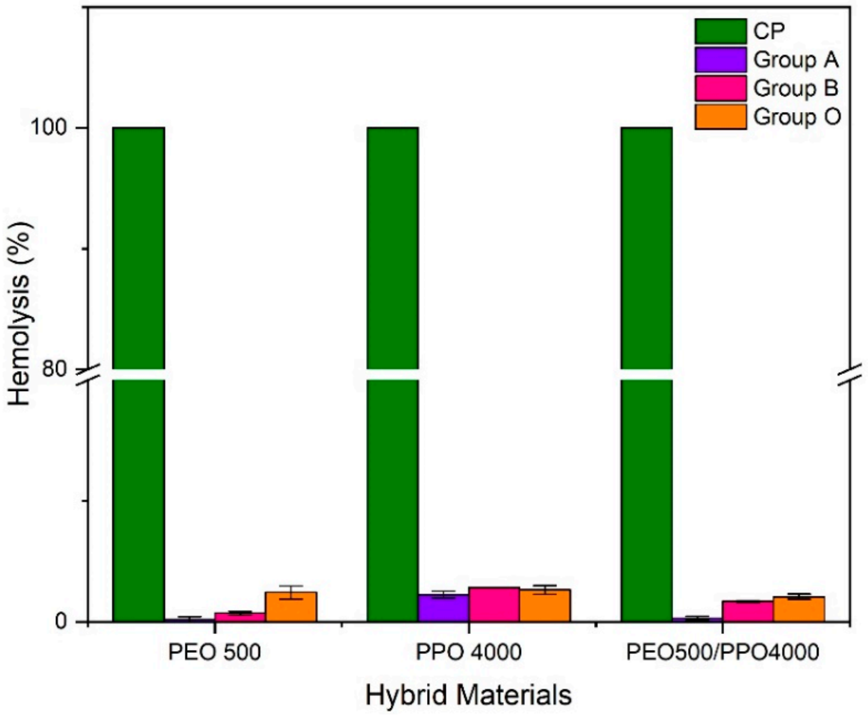

Figure S1. Comparison of the percentage of hemolysis between loaded 6% glucose membranes, ureasil-PEO500 and PPO4000, and blend of ureasil-PEO500/PPO4000 (50/50).
